# Error driven synapse augmented neurogenesis

**DOI:** 10.3389/frai.2022.949707

**Published:** 2022-10-28

**Authors:** Adam Perrett, Steve B. Furber, Oliver Rhodes

**Affiliations:** Department of Computer Science, The University of Manchester, Manchester, United Kingdom

**Keywords:** neurogenesis, synaptic activation, classification, regression, reinforcement learning, one-shot

## Abstract

Capturing the learning capabilities of the brain has the potential to revolutionize artificial intelligence. Humans display an impressive ability to acquire knowledge on the fly and immediately store it in a usable format. Parametric models of learning, such as gradient descent, focus on capturing the statistical properties of a data set. Information is precipitated into a network through repeated updates of connection weights in the direction gradients dictate will lead to less error. This work presents the EDN (Error Driven Neurogenesis) algorithm which explores how neurogenesis coupled with non-linear synaptic activations enables a biologically plausible mechanism to immediately store data in a one-shot, online fashion and readily apply it to a task without the need for parameter updates. Regression (auto-mpg) test error was reduced more than 135 times faster and converged to an error around three times smaller compared to gradient descent using ADAM optimization. EDN also reached the same level of performance in wine cultivar classification 25 times faster than gradient descent and twice as fast when applied to MNIST and the inverted pendulum (reinforcement learning).

## 1. Introduction

The brain possesses an impressive ability to acquire information and is able to readily apply it without a disconnect between learning and acting. An adult can be presented novel stimuli with associated labels and is immediately able to recall and manipulate these concepts. This is in part a consequence of the learning that has happened before (both in the life time of the human and on an evolutionary timescale) to extract general features, but it is also a consequence of the state in which information is stored in the brain. With time, through sleep and further training, memories can be consolidated and kept in a more general form (Stickgold, 2005) for future application.

One possible mechanism by which information can be stored in the brain is *via* neurogenesis, a process which continues throughout a lifetime (Spalding et al., 2013). Research has suggested that adult hippocampal neurogenesis plays a roll in learning and memory (Deng et al., 2010). Newborn neurons *de novo* grow axons and dendrites forming both efferent and afferent synapses enabling topological adaptation. The integration of neurogenesis in the hippocampus suggests an important role in learning and memory throughout a lifetime, which is missed from the majority of machine learning approaches.

Traditional Artificial Neural Network (ANN) training techniques, such as gradient descent, skip the initial acquisition of memories and their functional storage, and jump straight to building a generalized representation. These algorithms are examples of parametric models that allow an input-output mapping to be compressed into the parameters of a network. Owing to the universal approximation properties of artificial neurons with sigmoid activation (Cybenko, 1989), in theory, a statistical summary of any data can be captured in the parameters of an ANN. Learning usually begins with the weights of a predefined architecture being randomly initialized, the gradient of an error with respect to the weights is estimated and used to gradually move the network toward a position in the parameter space with reduced error. The random initialization can also have a bearing on the final optimum that will be converged upon due to a sensitivity to initial conditions. This is an incremental approach and requires averaging over many samples before a useful model can be elucidated.

Gradient descent uses data to build a statistical summary and incorporate it in the weights of the ANN. This leads to a condensed representation within the network and a generalized solution, assuming suitable architecture and hyperparameters. This condensed statistical representation leads to the black box nature of ANNs; their behavior cannot easily be investigated without evaluation using a test set. This can be problematic in cases with limited data where situations that must be accounted for are not part of the training data, such as different weather and lighting conditions for an autonomous car. It is also of importance in understanding what particular features are being selected for to produce an output, for example in medical diagnosis.

The general function of gradient descent algorithms applied to ANNs, such as Back-Propagation (BP), requires that the network be paused to perform an update. This is in part because of gradient descent often utilizing batch updates to smooth weight updates across multiple examples. There is also the computational demand of calculating gradients for all weights within the network and combining it with the produced error which can prove challenging during operation. Online learning could allow robots to explore an environment and adapt its behavior continuously, such as changing its gait to adapt to unexpected terrain or select new behavior after damage. Solutions exist to this such as the multi-compartment model of dendritic microcircuits (Sacramento et al., 2018) in which activity is simultaneously passed forwards and backwards to enable online updating of connection weights in a biologically plausible way.

Recent research has shown that the branching arms, dendrites, of L2/L3 pyramidal neurons possess non-linear activation functions capable of solving the XOR task (Gidon et al., 2020). They are not active with low level stimulus before a threshold level at which they begin producing activity. After a point, as input stimulus increases their activity decreases. This suggests some tuning of dendritic activity to a particular level of input activation. If that tuning can be adjusted to the level of inputs then the dendrites can be argued to have stored memory of those activations. This is a contrast to standard ANN connections which perform only linear transforms of inputs. There is evidence of dendritic dynamics and maintenance being involved in long term memory formation and cognition (Sutton and Schuman, 2006; Kasai et al., 2010), suggesting there is still far more untapped computational power within the biological brain yet to be harnessed by modern artificial intelligence.

Memory within neural networks often takes the form of Long Short Term Memory (LSTM) units (Sundermeyer et al., 2012; Greff et al., 2016; Bellec et al., 2018; Rao et al., 2022). They have shown great performance applied to time series data such as video and speech processing (Sak et al., 2014; Huang et al., 2015). They allow the storing of information in a way that can be trained *via* gradient descent which is ideal for traditional learning approaches in ANNs. External memory units have also been used in conjunction with neural networks (Weston et al., 2014; Graves et al., 2016). A powerful example is the neural Turing machine in Graves et al. (2014) which possesses memory locations which can be used to store and retrieve data. Their design allows training *via* gradient descent, as with LSTMs. However, due to their reliance on gradient based techniques they require large amounts of compute time to form and manipulate useful memories of the system. Deep neural networks have been augmented with episodic memory units in an attempt to solve the data hungriness of model-based neural network approaches. This is often in the form of a lookup table whose stored value is used in conjunction with a neural network trained with gradient descent (Lengyel and Dayan, 2008; Lopez-Paz and Ranzato, 2017; Lin et al., 2018). The addition of an episodic memory buffer enables quick acquisition of beneficial behaviors which can be exploited. This is exemplified in Blundell et al. (2016) in which a purely table based approach to episodic memory is used to solve Markov Decision Processes. The approach boasted fast learning capabilities as a result of being able to quickly exploit highly rewarding states. However, the episodic approach may be overtaken by parametric function approximators, such as DQN, in the later stages of training.

As mentioned earlier, neurogenesis has been suggested to play a role in learning and memory formation. However, neurogenesis has been a target of research within the machine learning community mainly with a focus on continual learning. In Draelos et al. (2017), this is displayed by first training an auto-encoder on a reduced number of Modified NIST (MNIST) classes using gradient descent. After adding the missing classes neurons are added to layers with a high reconstruction error and further trained with a reduced learning rate on non-new connections. This allows the network to adapt its architecture in response to the data and incorporate new information with mitigated catastrophic forgetting. Other examples of neurogenesis in literature often rely on starting first with a trained network and adding neurons *via* some method and further training the networks, such as in Mixter and Akoglu (2021) where neurons were added to a seed network using neuron activity as a selection metric. The algorithm grew the network displaying a reduced parameter size compared to standard approaches or methods employing pruning techniques with comparable MNIST performance. Martin and Pilly (2019) also start with a pre-trained network applied to MNIST. Following initial training a perturbed version of MNIST is presented and a genetic algorithm used to determine where new neurons should be added before being trained with stochastic variational inference to set the new weights. Parisi et al. (2018) apply neurogenesis to self-organizing maps with new neurons added to a pre-trained feature extractor layer and later trained with a Hebbian learning rule. Abolfazli Esfahani et al. (2021) use a seed network not trained on the desired task and instead took the first feature extraction layer of GoogLeNet trained on Places205 (Zhou et al., 2016), a place recognition data set. By utilizing the robust feature detectors of the first convolutional layer, particle swarm optimization was applied to control neurogenesis to create a corner detector.

There is very limited work on neurogenesis that does not rely on a seed network, the best example comes from Strannegård et al. (2019) in the paper *Lifelong Learning Starting From Zero*. They begin with an empty network and add neurons in response to errors, unlike the similar work of Eriksson and Westlund Gotby (2019) in which neurons are added for unrepresented states. Value nodes connected to neurons use Gaussian transfer functions to enable downstream neurons to be maximally responsive to particular inputs. The weights, biases, and Gaussian parameters are updated *via* backpropagation and gradient descent. Quick learning capabilities are displayed in contrast to networks trained with only gradient descent.

The algorithm explored in this paper, Error Driven Neurogenesis (EDN), leverages neurogenesis to enable online one-shot learning in a range of applications. It displays a rapid acquisition of new information, without the need for gradient calculation, in a biologically plausible way by modeling forms of neurogenesis and dendritic non-linearities. Starting from an empty network imbues it with no initialization bias and it is therefore only driven by the inputs it is presented and the context they are put in by the current performance of the network and how that effects the error generated. The paper is organized as follows: in Section 2 an overview will be given of EDN's design before discussing how it is applied to different domains in Section 2.2 and then contrasting it with similar algorithms in Section 2.3. Following this results are displayed in Section 3 and discussed in Section 4.

## 2. Methods

The current focus of machine learning research is often on the fine tuning of ANN parameters. An architecture is predetermined using expert knowledge to best suit a specific task and is gradually fed data to create a statistical model. This limits the general applicability of the network as a different topology may be required in a different domain and applying it to a new task can result in catastrophic forgetting, significantly hurting performance on the previously learnt task. It also does not enable the network to fine tune its structure in response to unexpected limitations, such as requiring more layers or more neurons to extract different features. Neurogenesis offers a potential solution to these problems by incorporating new neurons into a network. This can be done in a way that does not effect the previously learned information whilst allowing incorporation of new data. The algorithm explored in this work relies on three key principles: (1) errors drive learning, (2) synapses can be used to store information, and (3) neurogenesis facilitates information acquisition. When put in a learning scenario this combination leads to a modular network that grows in response to errors and stores information to mitigate these errors in its synapses. Code is available at: https://github.com/adamgoodtime/neurogenesis.

### 2.1. Error driven neurogenesis algorithm design

**Figure 1:**
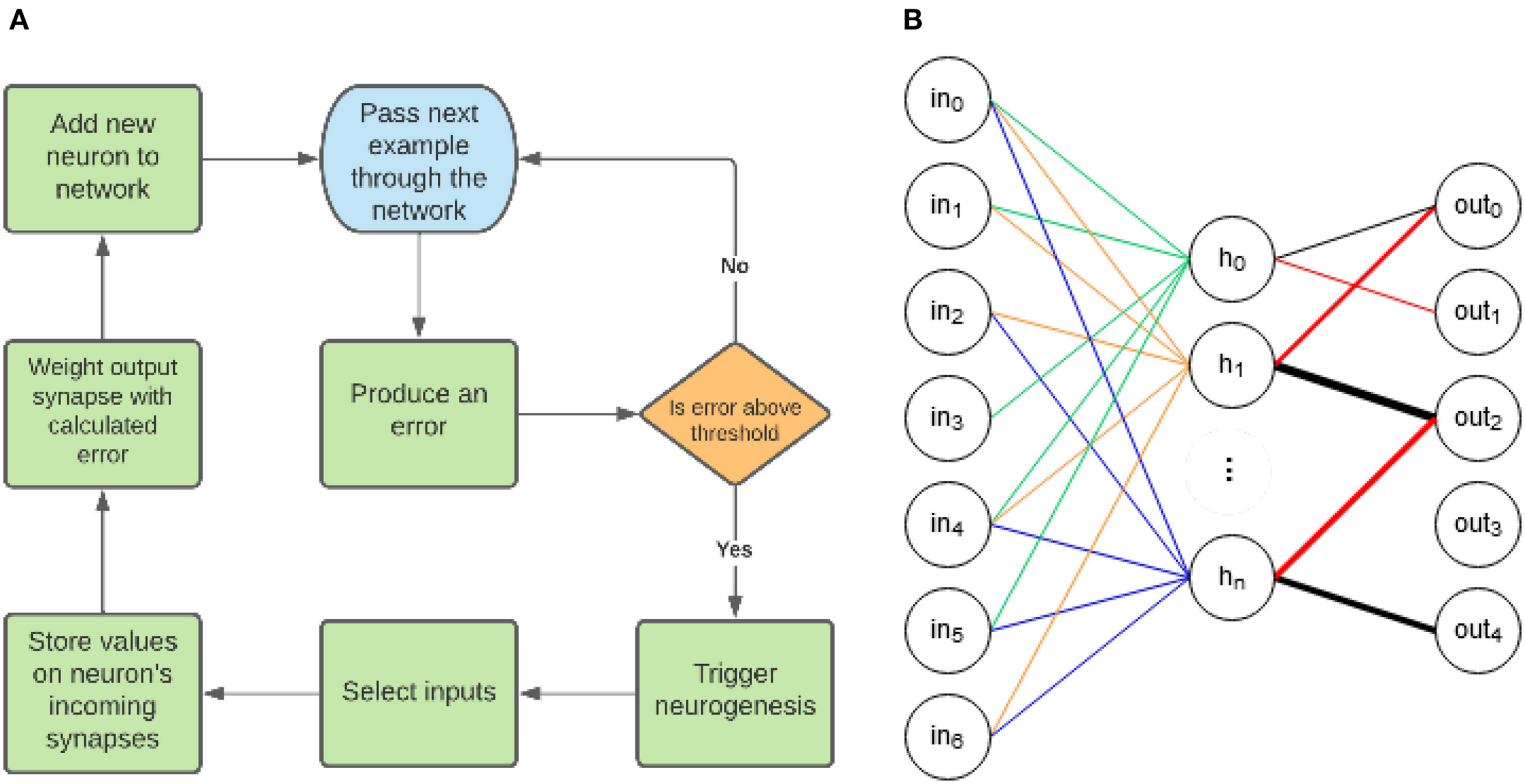
**(A)** The general structure of the EDN algorithm. First an input is presented which produces an associated error, if the magnitude of the error is above the error threshold then neurogenesis is triggered. Afferent synapses of the newborn neuron are selected and the values of the current inputs set the center of the kernel function on the synapses. The outputs whose error is above threshold are connected to the new neuron using synapses with weights proportional to the errors produced. **(B)** An example topology created with EDN. The different colors connected to the hidden neurons signify different sub-sampled input vectors which have been stored on the synapses; they all contribute equally to neuron activity. The color and thickness of connections between the hidden layer and the outputs displays that output connections are weighted and the magnitude can be negative or positive. The weight of these connections is determined during the training process by the error produced when neurogenesis is triggered.

An overview of EDN's operation can be seen in Figure 1A. There are three key elements to the EDN algorithm: error generation, neurogenesis and synaptic storage. First an input is presented to the network which in turn produces an output. The output is used to generate an error signal which is compared with a threshold to determine whether neurogenesis is triggered. Following the triggering of neurogenesis input values are selected to be stored, creating a neuron that is maximally active at the presentation of the same input. This newborn neuron is then connected to the corresponding outputs as determined by generated error, e.g., guessing a 3 when the class was 8 would connect negatively to the 3 and positively to the 8. This has now added a neuron to the network that upon seeing the same input of an 8 will inhibit the 3 and excite the 8. This process continues incrementally adding more information to the network each time the error is large enough. The repeated process of neurogenesis will build a network similar to Figure 1B. The following subsections will build on the overview of Figure 1 by first explaining how the network processes information and how that determines its representation. Next neurogenesis will be explained and how it makes use of the synapses to store information and alleviate errors.

#### 2.1.1. Network activation

The EDN network is composed of two elements, the neurons and the synapses. In contrast to traditional ANNs, the synapses contain the non-linearities, not the neurons. The synapse activation is a triangle kernel centered around a value *v* which is sampled from the inputs when neurogenesis is triggered and stored as a synapse parameter. The width of this kernel is controlled by the spread *s*. Equation (1) shows how an input, *x*, is transformed with the triangle kernel, *k*, to produce the synapse activation. This puts maximum synapse activation when the input *x* is the same as *v* with activation dropping the larger the separation until a difference of *s* at which it becomes zero. This is similar to the non-linear properties of dendrites discussed in Gidon et al. (2020) shown to be able to solve the XOR problem.


(1) $$\begin{array}{l} k\left(x\right)=\text{max}\left(0,1-\frac{\left|\left(v-x\right)\right|}{s}\right) \end{array}$$

(2) $$\begin{array}{l} \begin{array}{c} a_{n}=\frac{1}{N}\overset{N}{\underset{i}{\sum }}k\left(x_{i}\right) \end{array} \end{array}$$

Neuron activation, *a*_*n*_, is governed by Equation (2). The *N* inputs, *x*_*i*_, are passed through the triangle kernel, *k*, to produce the synapse activations. The synapse activations are averaged to produce the neuron activation without any weight term. This makes neuron activation a measure of similarity between the synapse parameter *v* (taken from previously stored input values) and the current inputs *x*. The measure of similarity is 0 − 1 because of the activation limits imposed by the function *k* and the averaging of the inputs. Synapses from the hidden neurons to the outputs also possess the non-linear activation shown in Equation (1) with *v* = 1 and the same value of *s* as the rest of the network. This acts to threshold neuron contributions to outputs to only the most similar detected features.


(3) $$\begin{array}{l} \begin{array}{c} a_{y}=\overset{N}{\underset{i}{\sum }}w_{i}k\left(a_{n_{i}}\right) \end{array} \end{array}$$

Output activation, *a*_*y*_, is controlled by Equation (3). Output values are calculated in much the same way as neuron activation with the main difference being the addition of a weight term *w* which is a function of the error (discussed in Section 2.2 for each task domain). The weight is calculated during training and modulates the contribution of each neuron, and therefore feature, to the output values. Also, activation is now the sum of the inputs rather than the average. As many hidden neurons can be connected to the outputs with only a fraction of them being active, the sum provides more information about relative difference between output activity.

**Figure 2:**
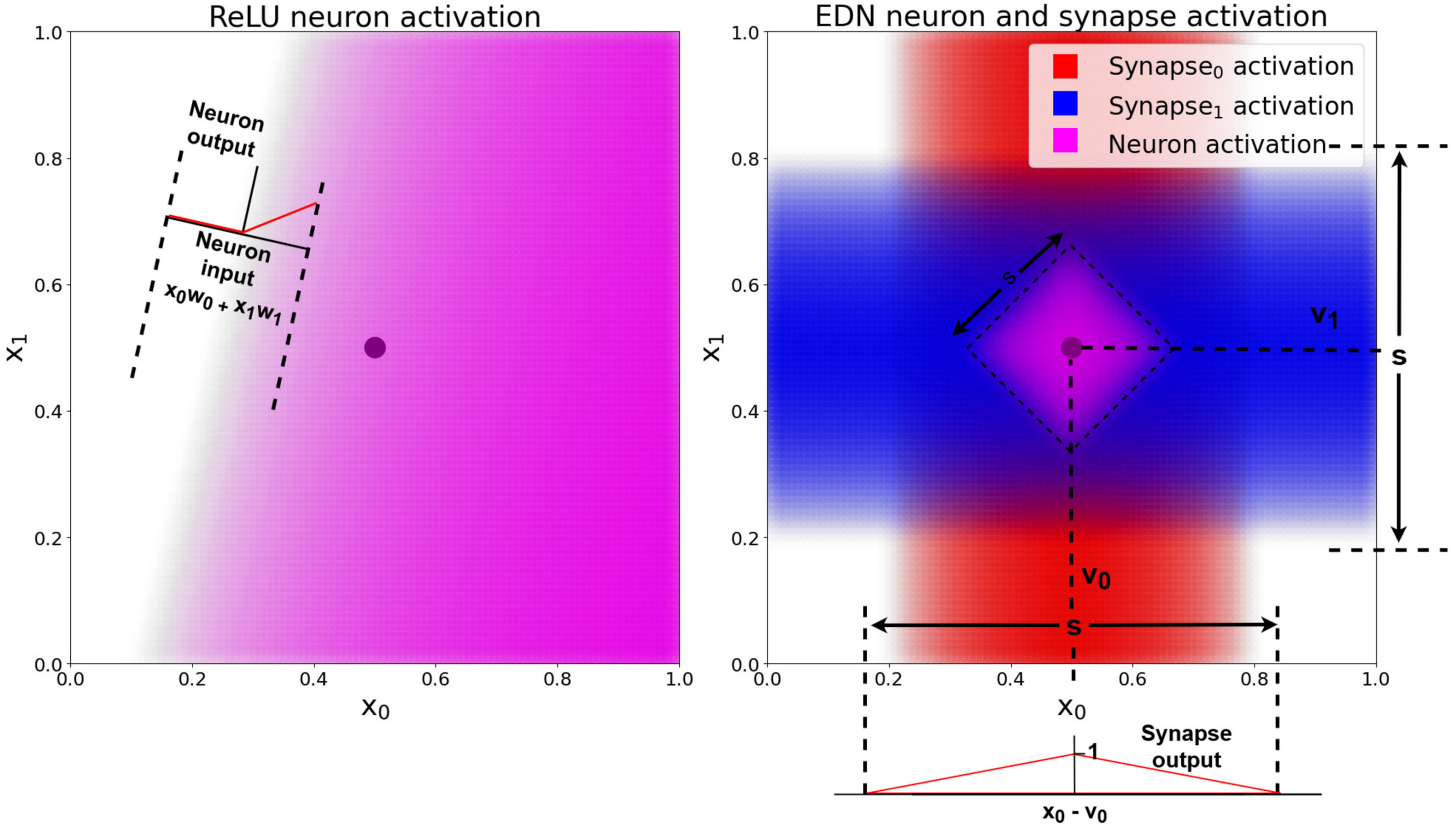
A comparison of a standard ANN neuron with ReLU activation and an EDN neuron's activation. The black dot is a single data point, the pink shaded area shows the output activation of the neuron in both cases, with a deeper color representing a higher level of activation. The red and blue shaded areas represent the level of activation of each synapse, which possess the triangle kernel shown in the bottom right, with spread *s* and centered at individual values of *v*. The EDN neuron's activity is the average of the incoming synapse activity and is also passed along an outgoing synapse using a triangle kernel with the same *s* value and *v* = 1. This output synapse acts to threshold neuron activity and create the bounded purple area in the input space in which the neuron is active.

A graphical 2D example of a single neuronal unit's activation (composed of synapses connecting inputs to the neuron and the neuron to an output) is shown in Figure 2. The EDN neuron's synapse activation can be seen to be highest around the values *v*_0_ and *v*_1_ and decreasing further from those stored values. As the neuron activation also passes through a synapse possessing a triangle kernel, the neuron activation is thresholded creating the diamond activation at the center of the two synapses peak activation, (*v*_0_, *v*_1_), which is narrower than the synapse activation.

#### 2.1.2. Network representation

A key difference in EDN compared to traditional ANN approaches is the way information is stored within a network. Neurons in a standard ANN take a linearly weighted sum of inputs and pass it through a non-linear activation function. This is analogous to drawing a hyperplane through n-dimensional space and having the neuron's activation relative to the distance from this plane (see Figure 2). By combining multiple neurons in multiple layers a complex high dimensional boundary can be created which determines a certain output for any input. The training of such a network typically requires the gradual acquisition of data to build a statistical summary of input-output mappings into the functionality of the network architecture.

Instead of neuron activity being relative to distance from a hyperplane, the activity of neurons in EDN are relative to distance from a data point. This removes the need for averaging over multiple instances as a single point can already say with confidence that the area around it is likely to share the same property. This is exemplified by Figure 2 where on the left a traditional ANN neuron with ReLU activation attempts to classify a single data point but without other data it becomes hard to say with any confidence where the boundary should be placed. On the right an ANN neuron's activation is shown with the neuron activation being limited to the area around the stored data point. The ReLU neuron will remain active infinitely far from the hyperplane. For this to be beneficial, generalization over many data points is required to create an appropriate model of the input-output mapping.

EDN neurons allow the instant acquisition of information and an adaptation of the network's model without disruption to previously learned information. The parameters retained within the model are never updated; information is only added never altered. In parametric versions of ANNs care must be taken when incorporating new information as error is transferred to the parameters (connection weights) of the network and can effectively overwrite previously learned knowledge. This is termed catastrophic forgetting and can limit the continual learning capabilities of ANNs as result of them being parametric models. Updating the model to include more information requires updating parameters which is where previous information is stored. The knowledge is being condensed into a limited number of parameters, therefore, changing any can alter the representation as a whole. Training a parametric model requires gradual tuning of parameters to ensure the model represents the data as a whole.

#### 2.1.3. Synapse creation

When neurogenesis is triggered the current input values are retrieved to become the *v* parameter for synapses connecting to the newborn neuron. They form the afferent synapses of the newborn neuron creating a neuron which is maximally active when presented with the same inputs as were just stored. Subsets of inputs can be selected to be the incoming synapses of the neuron. This moves the neuronal representation from an input-output mapping to a feature-output mapping. Efferent synapses of all hidden neurons have a uniform *v* equal to 1, in contrast to the afferent synapses where *v* is set by the values of the inputs. Because the synapses still possess a triangle kernel this acts to threshold and amplify output contributions of neurons which are receiving the most similar inputs to their stored values. Neuron contributions to outputs is further modulated by error, discussed next.

#### 2.1.4. Error integration

The algorithm uses neurogenesis to mitigate errors produced by the network. Neurogenesis is only triggered if the magnitude of the error is above the error threshold *E*_*th*_. The way in which error is produced and integrated into the network is task specific and will be elaborated in Section 2.2. A general way to view it is to compare it to how weights are updated in gradient descent. The sign of the gradient indicates which direction the weight should be altered, more or less positive, to reduce the error. You subtract the gradient from the weight to move the weight in the opposite direction of the gradient of the error with respect to the weight. This update can be broken into two components: a magnitude and a direction. In EDN the magnitude is incorporated into the *w* parameter of efferent synapses of hidden neurons to outputs. The direction is governed by the inputs stored in the afferent synapses' *v* parameter. This in essence makes each neuron an individual update to overall network performance.

**Figure 3:**
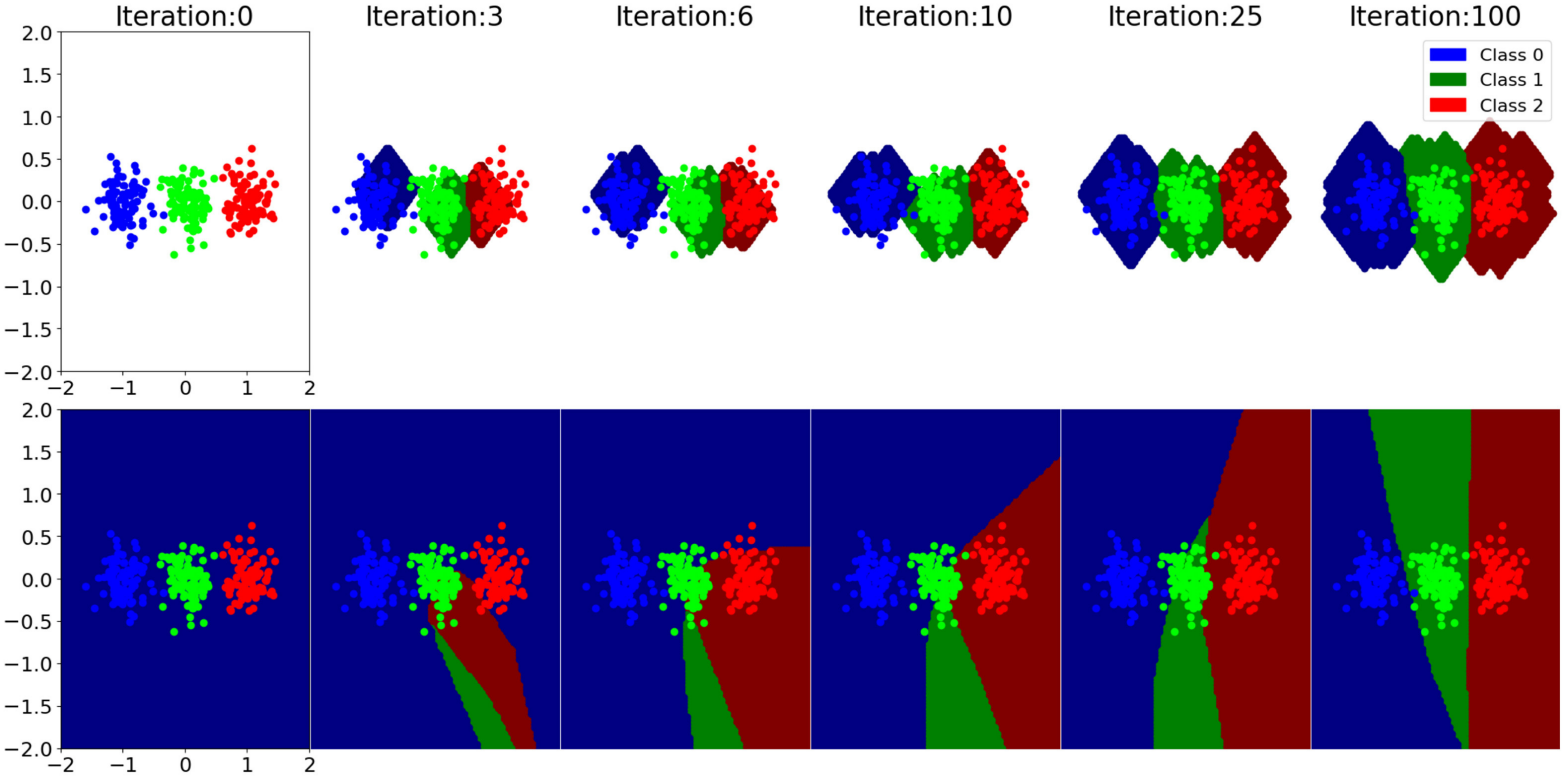
A selection of training steps of EDN **(top)** and an ANN trained with gradient descent **(bottom)** are shown for a toy classification example. Each training iteration is a single input presentation with the boundary being displayed after the network has been updated to incorporate the training error. The data set is composed of three classes: blue, green, and red. Class boundaries are drawn in a darker shade than the data points showing what output each model would associate to that point in space. The white area around the EDN plots indicates that there are no output activation at that point in the input space, meaning all outputs are zero and, therefore, no class is chosen.

A comparison of learning dynamics between gradient descent and EDN can be seen in Figure 3. It is a 2D toy example composed of three classes: red, green, and blue, with all points being the training data. Decision boundaries are drawn for each class at each training step. At the start EDN has no output representation and therefore makes no guess about which output belongs to what point in the input space, this can be seen as the white area around the points. The network initialization of gradient descent produces a bias at the beginning of training creating an output for all points in space. This is a consequence of the neuron activation that is active infinitely far from the hyperplane drawn by the incoming synapse weights, although they have no bearing on the data at this stage. As training progresses the class boundaries drawn by gradient descent shift and begin to match the data. It is also seen that EDN's storage of a few data points enables the classification of many other data points immediately. This trend continues with gradient descent gradually matching the input data until a general approximation of data distribution is converged upon. EDN converges to a general boundary surrounding the classes, although, there remains no activity far from the data points. If subsamples of inputs were taken neuron activity could persist along selected dimensions, however, with this example all inputs are selected to be a part of the new neuron. The network trained with gradient descent is active far beyond the training data, although there is no way to be certain those extrapolations are correct.

#### 2.1.5. The neuronal unit

The network in EDN is a collection of independent and modular neurons. Each one has a set of input synapses that are more active the closer the input activity is to their stored *v* value. A neuron's contribution to the outputs is proportional to the output error produced when that neuron was created. Combined, this creates a modular neuronal unit that attributes a specific output value to inputs similar to its stored *v*-values. The more similar the input the more confident the neuron is in the expected output. En masse this creates a network that, through training, has an expectation of the correct output for all points within a certain distance of the saved features.

The storage of information on the synapses allows neurons to be investigated and input-output mappings to be extracted from the network. The values of *v* on the afferent synapses store the inputs and the efferent synapses indicate which outputs those inputs correspond to. When subsets of inputs are selected this enables feature-output mappings to be extracted. Parametric models do not possess this ability as the knowledge is condensed into a set number of parameters. Evaluating the model requires querying it with input and observing the output. Investigating output response to inputs is important to be able to accurately examine performance, however, being able to dissect a network and determine what constituent elements compose the behavior as a whole enables fine grained inspection and conclusions to drawn beyond the data presented to the model.

### 2.2. Task specific alterations

For all tests input values are normalized between 0 and 1 by subtracting the minimum and dividing by the range before training. An error threshold, *E*_*th*_, determines the minimum absolute error that is required for neurogenesis to trigger. Only outputs with a magnitude of error greater than *E*_*th*_ will form connections with the new neuron.

#### 2.2.1. Classification

For the classification tasks the error is generated by subtracting the estimated classification (softmax of the output values), y*, from a one-hot encoding of the correct labels, *y*. This gives positive error for outputs which were not large enough and negative error for outputs which were too high. As shown in Equation (4), this error value, *E*, becomes the weight of the connection from a new neuron *j* to the respective output *i* if neurogenesis is triggered.


(4) $$\begin{array}{l} w_{ji}=y_{i}-y{*}_{i}=E_{i} \end{array}$$

#### 2.2.2. Using surprise to guide input selection in classification

During classification, when neurogenesis is triggered a record of the input values stored on afferent synapses is added to an expectation for the associated class. Similar to the way regression values are calculated and saved, discussed in Section 2.2.3, the stored values of *v* and their inverses are collected from new neurons creating a combined representation of the classes.

When neurogenesis is triggered the expected output for each class, *e*_*y*_, is multiplied by the softmax activation values of their respective outputs, *a*_*y*_, and summed across outputs creating a combined expected output, *e* (see Equation 5). The activity modulated expectation and the input values, *x*, are compared for each individual input, *i*, to create the input surprise *s*, as shown in Equation (6). Inputs with a surprise greater than the surprise threshold, *s*_*th*_, are selected to form synapses of the new neuron. When there is no expected value of an input, such as at the beginning of training, the input is selected by default.


(5) $$\begin{array}{l} e=\overset{outputs}{\underset{y}{\sum }}a_{y}e_{y} \end{array}$$

(6) $$\begin{array}{l} s_{i}=\text{abs}\left(e_{i}-x_{i}\right) \end{array}$$

#### 2.2.3. Encoding continuous values

Classification is a mapping from inputs to a discrete label but many possible input output mappings do not map to discrete outputs. Regression tasks involve mapping inputs to continuous values and, therefore, require a different formulation to work with EDN. Neurons within EDN attribute a specific input to a specific output error. In classification this is straightforward as neurons can be directly connected to the outputs associated with the error produced. In regression this is less simple as outputs can take any value within a continuous range. The output connection has to be able to point at a specific position on a scale. It also has to be able to point with a certain magnitude to indicate similarity with stored inputs (neuron activity) and the associated error created during neurogenesis (output weight).

**Figure 4:**
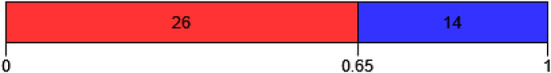
An example of how output values of 0 and 1 are combined to create a position on the regression scale. As described in Equation (7) the output value for the real part of the scale (red) is divided by the total of the real and inverted (blue) outputs to create the position on the regression scale, in this case $\frac{26}{26+14}=0.65$. This value is then scaled to the full range of regression values possible in the task to produce the estimated regression value of the input.

These challenges are solved by splitting each regression value into two components, one for the real value on a scale and one for the inverse value. The scale is from 0 to 1 and is rescaled to fit the full range of output values possible for the specific task. A newborn neuron is connected to both the real and the inverse outputs. The total activity of the network's contribution to both outputs is combined to estimate the regression value. This is exemplified in Figure 4 where a value of 0.65 on a scale from 0 to 1 is encoded as 26 for the real output and 14 for the inverse. Equation (7) is used to retrieve that position on the scale, where *x* is the value, *r* is the real valued magnitude and *i* is the inverse magnitude. This splitting enables the activity level of a neuron to not effect the estimated output value and instead become a measure of confidence in the value. Multiple neurons' activities can also be added together and modified by individual output weights to create a weighted average of regression estimates.


(7) $$\begin{array}{l} x=\frac{r}{r+i} \end{array}$$

During operation all output values are bounded from 0 to 1 and used to calculate outputs as shown in Figure 4. The range of possible regression values is used to calculate what the minimum and maximum values should be when translating back and forth between a scale from 0 to 1 and actual regression values. They are scaled to the full range of regression values to calculate the mean squared error for a given input and 0–1 when represented in the network. This error, *E* then becomes the connection weighting from a neuron to the real, *w*_*r*_, and inverse, *w*_*i*_, outputs with the ratio between the two encoding the position in the output range associated with that input. This is shown in Equation (8) and Equation (9) where *x* is the current regression value for that input that needs to be stored on the neuron, *min* is the minimum possible regression value and *max* is the maximum possible value. This attributes a particular input with a particular regression value and weights it by the error produced by the network.


(8) $$\begin{array}{l} w_{r}=E\frac{x-\text{min}}{\text{max }-\text{ min}} \end{array}$$

(9) $$\begin{array}{l} w_{i}=E\left(1-\frac{x-\text{min}}{\text{max }-\text{ min}}\right) \end{array}$$

#### 2.2.4. Reinforcement learning

Classification and regression are learning paradigms where a model is trained with data in which all inputs are given an output label, either discrete or continuous. Reinforcement learning scenarios do not have access to such information and must make connections between actions taken through time and their eventual reward state. In EDN, actions are taken greedily with the output with the highest value used to determine the action performed. Random actions are taken when all outputs are equal. There is no correct label due to the reinforcement learning nature of the task, therefore, errors are simulated by inhibiting the last *m* timesteps before a failure. This is in an effort to reduce the chance of the action associated with a negative behavior happening in the future. The most recent timestep is given an error of −1, then decreasing by $\frac{1}{m}$ until the *t* − *m*^*th*^ timestep after which no more neurons are formed. This generates *m* new neurons for each failure with the neurons connected to the output chosen at that particular timestep. Equation (10) shows how output weights are generated. Only the *ith* output corresponding to action *i* being chosen, *a*_*i*_, at timestep *t* forms a connection with the new neuron.


(10) $$\begin{array}{l} w_{i}=-a_{i}\left(1-\frac{t}{m}\right) \end{array}$$

#### 2.2.5. Neuron reinforcement and deletion

Reinforcement learning presents the challenge of associating actions with rewards. Unlike classification and regression there is not a specific input output mapping to learn *via* supervision. Negative reinforcement is captured in the error driven neurogenesis. In the inverted pendulum task a positive reward is given at each timestep the pole is balanced. This signal is passed into the network and used to keep track of each neuron's contribution to network performance. A neuron's individual reward value, *R*, is first initialized to the average of the network's reward, starting at zero for the first neuron, to allow time for new neurons to be evaluated. The equation for updating the neuron's reward, *R*, at each timestep can be seen in Equation (11) where *r* = 1 is multiplied by the activity of the neuron, *a*_*n*_, and low-pass filtered with a τ value of 0.9999.

To remove unrewarding behaviors, and keep the best input-action mappings, neuron deletion is used in conjunction with neuron reward to prune the network of the least productive neurons. This is done when the number of neurons in the network goes above a set limit. When a neuron needs to be deleted the neuron with the lowest accumulated reward is selected to be removed. This aids in capping network size and has the added benefit of aiding speed of convergence.


(11) $$\begin{array}{l} R\left(t+1\right)=\tau R\left(t\right)+\left[\left(1-\tau \right)ra_{n}\left(t+1\right)\right] \end{array}$$

### 2.3. Related algorithms

While EDN shares attributes with ANNs and gradient descent it also shares commonalities with other learning algorithms. The methodologies discussed below are similar in formulation to EDN and are introduced to highlight the similarities to and differences from related algorithms. They do not have the same breadth of applicability as backpropagation and are therefore not used for comparison in the results.

#### 2.3.1. K-nearest neighbors

A similar non-parametric algorithm that leverages the data points to form the function output of the model is K-Nearest Neighbors (KNN) (Fix and Hodges, 1989). All the training data forms the model with the K closest training data points performing a majority vote to determine the property of an unseen point. Careful tailoring of K is needed to ensure appropriate classification (Cover and Hart, 1967). EDN does not require the defining of a K with the training process selecting and weighting individual data point's contribution to the combined model output. The thresholding of output synapse activity puts a limit on how many neurons will contribute to the prediction. The synapse thresholding also alters the way in which distance between data points is measured; if two input vectors are similar in all but a small number of inputs which are very different then Euclidean distance between them can become large. In contrast the thresholded synapse activity will put a cap on the distance between variables allowing the vector as a whole to still be considered similar to stored values if a minority are very dissimilar. This puts an emphasis on the feature-output mapping rather than the input-output mapping. Only if an error is produced will this assumption be adjusted and neurogenesis triggered to alter the belief. This mechanism in combination with input subsampling puts a stronger emphasis on the features and their relation to network error rather than the data points as a whole.

#### 2.3.2. Radial basis functions

Radial Basis Function (RBF) networks share a similar topological design to EDN. They both have a single layer of neurons whose activity is distant-dependent from their centers to the input point with the peak at zero distance. However, EDN uses a triangle kernel on each synapse rather than an absolute distance between two points. The neuron then becomes a measure of distance between features rather than points in *n*-dimensional space. This emphasis enables exploration of different feature combinations and their contribution to performance and allows neurons to have broader applicability outside their local area.

When designing an RBF network, once a kernel has been decided, often Gaussian, there are four main parameters of RBF networks: number of nodes, center of nodes, radius of the function, and the weights of the RBF outputs (see Dash et al., 2016 for a survey of RBF networks). Common training methods for determining the centers of nodes involve a clustering of the data points, following this an iterative process of calculating the radius and weights of each radial basis function reduces some objective error and guides the network toward a local optimum. EDN avoids the need for gradient descent and tuning of neuron parameters by computing error on the fly, and combining this with the current input to adjust the network as a whole. This avoids the need for network parameter optimization, however, EDN will end up with more nodes within the network compared to a typical RBF network.

#### 2.3.3. Kernel density estimation

Another algorithm with commonalities to EDN is that of kernel density estimation (KDE) (Silverman, 1998). By applying a kernel to individual samples an approximation of the data distribution can be established. This technique finds its main use within establishing a probability distribution over a geographical area. Applying a kernel to samples within a space allows inference to be made about the area surrounding the samples. This can even be extended to the time domain as in Nakaya and Yano (2010) where the temporal element of crime statistics is used to create an estimated crime density in Kyoto in both space and time.

The main difference between KDE and EDN is the uniformity of kernel contribution to the overall output. In KDE each data point is part of a random distribution and the objective is to combine those samples to estimate that distribution. Data points are all considered of equal importance and specific inputs are not subsampled to extract different features. With EDN the data points are not all considered uniformly and are only added to the network if they are not currently captured by the model, as determined by the error during operation. This pivot toward an error driven distribution removes the need to store all data points in a model and also enables application to a wide variety of machine learning domains. The subsampling of inputs enables useful features to be extracted in contrast to all inputs contributing equally.

## 3. Results

Results are shown for the individual tasks comparing EDN against Gradient Descent (GD) using the ADAM optimizer. Following this a parametric analysis of EDN is given using the wine data set to explore the sensitivity and influence of different parameters. Parameter values for both EDN and GD were found *via* grid search for each task individually and given below. Parameters for both were selected to optimize primarily for speed of learning without hurting the stability of performance.

### 3.1. Wine—classification

**Figure 5:**
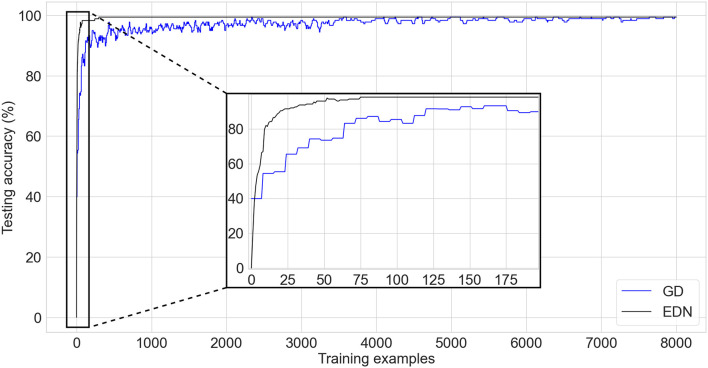
A comparison of testing accuracy during training on the wine cultivar classification task of EDN (black) and an ANN trained *via* gradient descent using ADAM optimization (blue). An inset is shown for the first few iterations of training to display the initial emptiness of the EDN network producing no output and the initialization bias of the ANN network already achieving testing accuracy equivalent to random choice. The fast acquisition of information in EDN allows it to overtake the testing accuracy of GD before the first batch update is done at the 8th training instance.

The UCI wine data set (Dua and Graff, 2019) is a standard test classification benchmark comprised of 13 inputs and three possible output labels determining the cultivar/type of wine. There are 178 training examples with a class distribution of 59, 71, and 48 for each class, respectively. A stratified K-fold cross-validation of K = 10 and test set size of 10% is used to evaluate the performance of the algorithms. A comparison of performance between EDN and a network trained with GD can be seen in Figure 5. After every training update networks are evaluated on the entirety of the test set and the average across each fold is displayed. The EDN parameters used were: *s* = 0.4, *E*_*th*_ = 0.1, *s*_*th*_ = 0.05. GD used a learning rate of 0.03 with ADAM optimization, a batch size of 8 and a network with a single layer of 200 neurons, a network of similar size to the final number of neurons created in EDN training was selected for comparison.

The bias created by the initialization of the ANN can be seen in the performance starting at an average testing accuracy of 39.9%. This roughly mirrors the testing accuracy of random output selection given the class distribution of this task. In contrast, EDN begins with an empty network and, therefore, starts with no initial bias. This results in the network not being able to make any initial guesses and beginning with a testing accuracy 0%. The training process adds neurons to the network, rapidly increasing the testing accuracy and surpassing the performance of the GD after four samples, this is before the first batch update of GD has even been calculated. It can also be seen that learning *via* GD causes fluctuation in testing accuracy which stabilizes with time. EDN's performance displays less variance between training examples with a final performance of 99.4+% reached in 298 iterations using 216 neurons. A few thousand training examples is required by GD before testing accuracy converges toward a performance of 99.4%. This is over 25 times slower than EDN with testing accuracy still not completely converged. Neuron count continues to increase in EDN with testing accuracy remaining constant throughout the remainder of the training. Early stopping could have been used here to limit network growth, however, it is continued to display the lack of overfitting present after convergence.

### 3.2. Auto-mpg—non-linear regression

The auto-mpg regression data set (mis, 1993) consists of 398 cars with 9 attributes such as number of engine cylinders and horsepower. The aim is to process the attributes and output the miles-per-gallon of the car. K-fold cross validation of K = 10 is used to evaluate performance. The results shown are the average testing accuracy across all folds. The EDN parameters used were *s* = 0.4, *E*_*th*_ = 0 and all inputs were selected by each neuron. Two different configurations are shown for GD to display how batch size and learning rate can effect learning. A single layer of 1024 hidden neurons is used as this resulted in the fastest and most stable learning using ADAM optimization.

**Figure 6:**
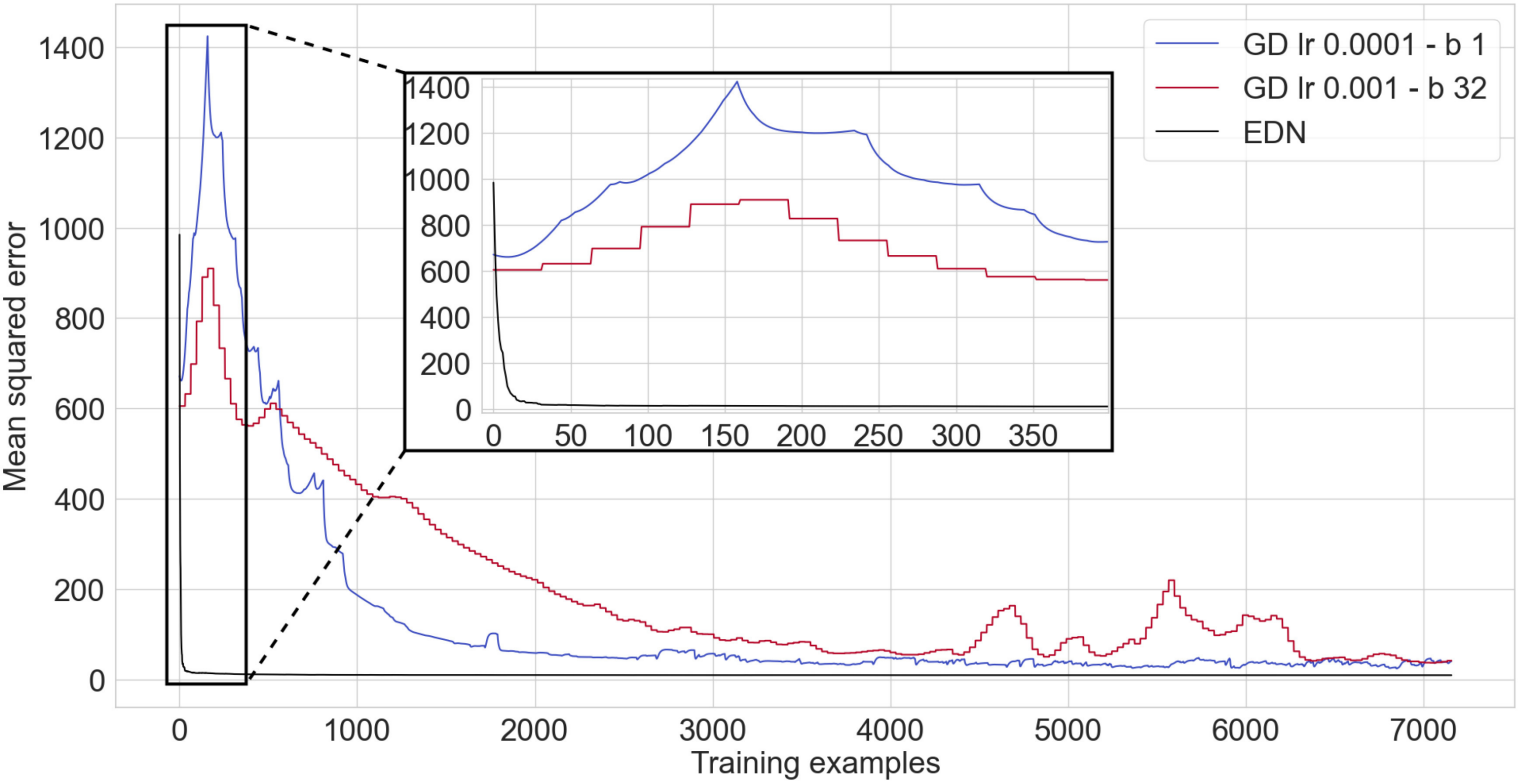
A comparison of an ANN trained *via* gradient descent using ADAM optimization (red and blue, with lr being the learning rate and b being the batch size) against EDN (black) applied to the auto-mpg non-linear regression task. A zoomed in inset is given to display the learning curve of EDN. Batch sizes and learning rates were chosen which gave the fastest convergence in gradient descent.

Mean squared error (MSE) is calculated over the whole testing set after each presentation of an input or batch in the GD cases where the batch size is greater than one. A comparison between EDN and GD on the auto-mpg data set can be seen in Figure 6. EDN begins with its MSE above the ANN's, however, it rapidly acquires enough data points to surpass any converged MSE achieved by GD. GD achieved a minimum MSE of 24.9*mpg*^2^ with a batch size of 1 and 32.9*mpg*^2^ with a batch size of 32 during the 20 epochs of training. EDN surpasses the minimum achieved by GD with an MSE of 23.2*mpg*^2^ after 29 training examples (creating 29 neurons) and an MSE of 12.2*mpg*^2^ at the end of the first epoch (creating 358 neurons as *E*_*th*_ = 0 so every sample creates a neuron). The error continues to be fine tuned during the proceeding epochs until a converged MSE of 10.25*mpg*^2^. If it assumed that GD reached its best performance at 4,000 training iterations (even though performance has not converged yet, especially for the batch size of 32) this makes EDN over 135 times faster than GD to achieve the same result. With further training EDN also converged toward an MSE almost three times smaller than GD.

Gradient descent takes considerably longer to produce the same level of performance. EDN can achieve a faster acquisition of appropriate regression values as neurogenesis can instantly store values. This mechanism enables any data points close to this value to be attributed a similar value which is often close to its true value in regression. EDN is also not effected by the non-linear nature of this regression problem compared to GD. As described in Section 2.1.2, the combination of synapses and neurons in the GD ANN draws a hyperplane through the input dimensions with a neuron's activity being relative to the distance from this plane. This makes producing a smooth output value across the input space difficult for GD to achieve, especially in non-linear regression.

### 3.3. MNIST—visual classification

MNIST hand-written digit recognition is a common benchmark used in visual classification. It is comprised of the numbers 0–9 discretized into a 28 × 28 grid with grey-scale pixel intensity from 0 to 255. The data is split into a training set of 60,000 digits and a test set of 10,000. The dimensionality of the inputs is far greater for this task than those explored previously and, therefore, an appropriate sampling of the inputs is important to aid performance and reduced the number of parameters in the network. EDN parameters used for experiments unless otherwise specified were *s*_*th*_ = 0.4, *E*_*th*_ = 0.1, and a kernel spread *s* = 0.4. For EDN with random input selection the number of input synapses per neuron was limited to 150, which produced the best performance and is inline with the average number of synapses selected per neuron with using *s*_*th*_ = 0.4. The ANN trained with gradient descent with ADAM optimization used 1024 neurons with ReLU activation, a learning rate of 0.001 and a batch size of 64.

**Figure 7:**
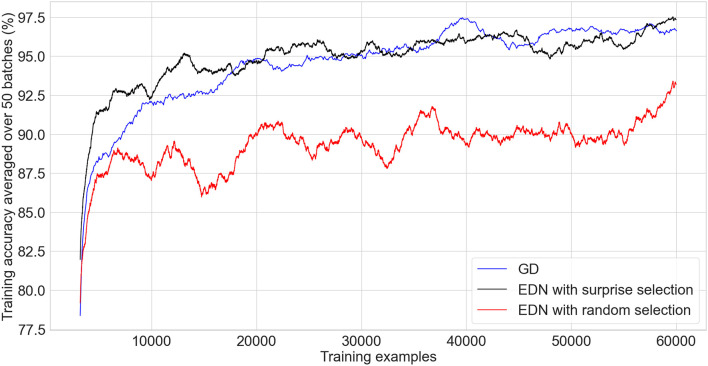
A comparison of an ANN trained in tensorflow using ADAM optimization (black) against EDN with surprise driven input selection (blue) and random input selection (red) applied to the MNIST classification task. A batch size of 64 is used to train the ANN and the moving average of the last 50 batches is used to calculate the running training accuracy. EDN training accuracy is the moving average of the last 3,200 (50*64) training examples as it does not perform batch updates. Training accuracy is used as, since these are the results of the first epoch, non of the data has been seen before and therefore it is equivalent to testing accuracy.

The graph in Figure 7 compares the performance of EDN against GD. A running average of training classifications is used to enable a fine grained comparison without needing to evaluate over the test set after every training example. As the comparison only shows the first epoch none of the training examples have been seen before and therefore it is equivalent to testing accuracy. As with the previously discussed tasks, a fast acquisition of information allows EDN to reach a higher level of accuracy faster than GD in the initial stages. EDN reaches 90, 92.5, and 95% accuracy after around 4,300, 6,250, 13,000 iterations, respectively, whereas GD takes around 7,200, 12,000, and 25,000 making EDN almost two times faster. When EDN uses random input selection the network's ability to collect the most pertinent input information is hampered resulting in slower learning and a maximum accuracy of around 93%. This highlights the importance of surprise driven input selection to guide the network toward the inputs representing information not currently captured by the model.

After one epoch the testing accuracy of EDN is 96.3% with surprise selection and 90.93% with random input selection. GD achieves 96.7% putting surprise selection at comparable levels of performance after seeing the entire training set. EDN's testing accuracy does not increase considerably with further training, gaining only another 0.5% after another epoch and little after that. The parameters for GD were chosen to produce the fastest, stable learning meaning that continued training did not push testing accuracy to the 98%+ seen with state-of-the-art training methods, however, further epochs continued to improve performance up to around 97.5%. These experiments display how EDN is able to acquire information quickly and store it in a functional way that can be applied to the task, however, the ability of GD to perform slight alterations of parameters allows continued refinement of the model which is not possible with EDN's use of a uniform kernel.

### 3.4. Visualizing the expectation

**Figure 8:**
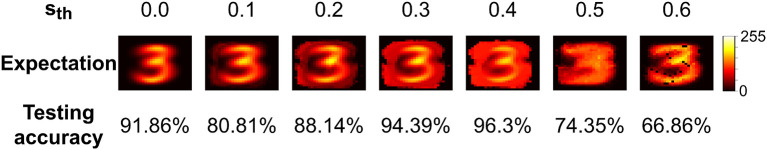
A visualization of the expectation for the class 3 retrieved from the parameters stored by EDN during training on MNIST. A range of surprise thresholds, *s*_*th*_, are given to show how this effects the stored values and the subsequent effect on testing accuracy after a single epoch through the training set.

Using the input surprise method outlined in Section 2.2.2, an expected output can be generated for each class. The expectation is generated by an unweighted combination of the input values stored in the network's *v* parameter for each class. In Figure 8, the effect of the surprise threshold, *s*_*th*_, on the expectation and performance after one epoch can be seen. When *s*_*th*_ = 0 this corresponds to all inputs being saved when neurogenesis is triggered which leads to a well defined expectation of a 3. As *s*_*th*_ is increased the network now begins to use the expectation of a 3 to guide input selection.

At low values of *s*_*th*_ the performance suffers as many inputs are above threshold, and therefore form synapses with the new neuron, but the most similar inputs do not, resulting in a neuron with an unrepresentative feature-output mapping. When combined they create a good expectation of the class, as can be seen in Figure 8, but individually their feature detection suffers. As the threshold increases the neurons begin to select inputs with a greater emphasis on the features of the input that make it different from previous presentations. This leads to a greater diversity and selectivity of feature-output combinations captured by the network. The best performance can be seen when *s*_*th*_ = 0.4 and this comes when the expectation is more evenly distributed around the input. This broader expectation allows only the most different inputs to be selected without too much focus on saving what is already captured by the model. When *s*_*th*_ increases beyond this point performance drops considerably as now the threshold is too high for a representative sample of the class to be captured. At *s*_*th*_ = 0.6 it can be seen that very few samples are taken as there is pixelation from the lack of samples averaging out the expectation.

### 3.5. Visualizing the receptive fields

**Figure 9:**
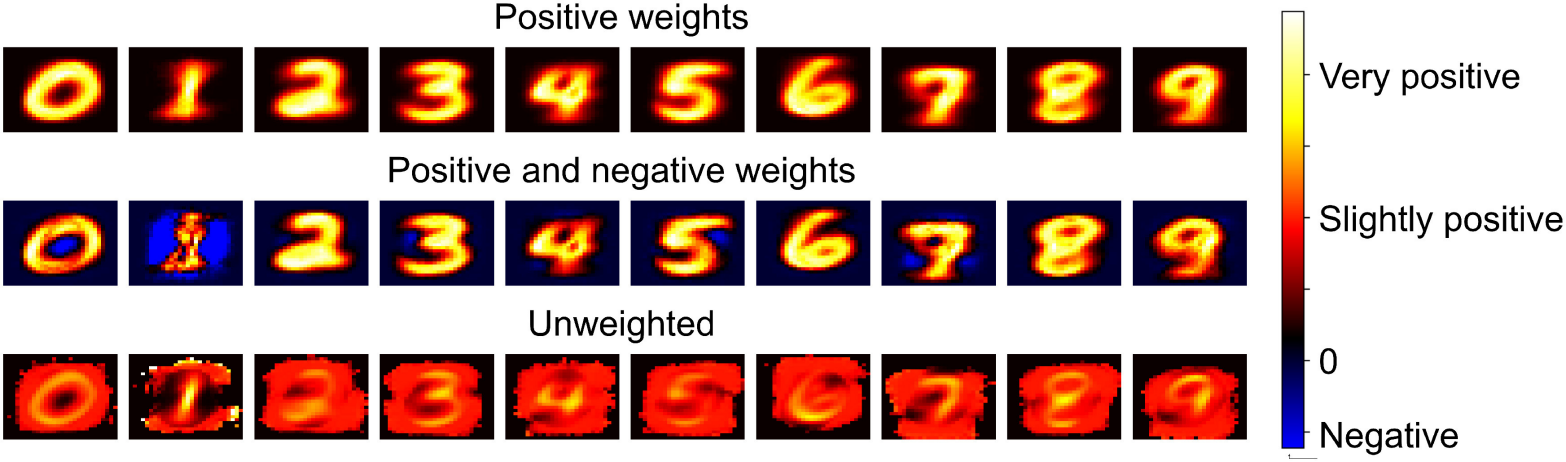
The values of *v* stored on each neuron's incoming synapses form a record of the inputs which were captured by EDN during training. The weight on the synapse connecting the neurons to the outputs enables the receptive field of each class to be determined by multiplying the stored values by their neuron's associated weights. When taking the positively weighted neurons of each class and multiplying their stored *v* values by their associated output weight you create the first row of the plot, this forms an weighted expectation of each class. The second row is generated by also including the negatively weighted neurons connected to each output, showing the average receptive field of each class. The bottom row is an unweighted combination of the input synapses which are instances of each class.

The previously explored expectation was an unweighted collection of each class's stored values. To examine what each class is sensitive to the weightings of each neuron are included. In Figure 9, the receptive fields are extracted in the same way as described in Section 2.2.3 with the stored values of *v* on the synapses and their inverses being multiplied by their associated weight, the weight from the neuron to the output. The top part of Figure 9 displays the weighted sum of the neurons which connect positively to the respective outputs, forming an average representation of each class. When negative weights are also included the full receptive field for each class can be seen. Prominent examples of inhibition can be seen for class 0 and 1 where there is strong negative weighting at the center of the 0 and to the sides of the 1. This technique shows it is straightforward to extract the information present in the network and evaluate what each class is responsive to. It is possible that with some form of clustering that subdivision of each class could also be extracted, such as sevens with and without a line crossing their middle. Here just the average receptive field is presented.

### 3.6. Inverted pendulum—reinforcement learning

Networks are connected to the TensorFlow gym environment (Brockman et al., 2016) cartpole_v1 to test performance. The task is to keep a pole balanced on a cart without the cart moving too far from the starting position or the angle of the pole moving too far from the vertical. The maximum balance time is 500 timesteps with the task being considered solved if the average balance time over the last 100 trials is over 475. A slightly broader kernel proved effective in this task resulting in *s* = 0.6 being chosen. A memory length, *m*, of 10 was sufficient which means the last 10 timesteps are classified as a failure and each trigger an individual neurogenesis step. All inputs are selected by each neuron. An ANN actor-critic model is trained using GD, a learning rate of 0.003 with Adam optimization, 128 hidden neurons (more did not improve performance) and a gamma value of 0.99.

**Figure 10:**
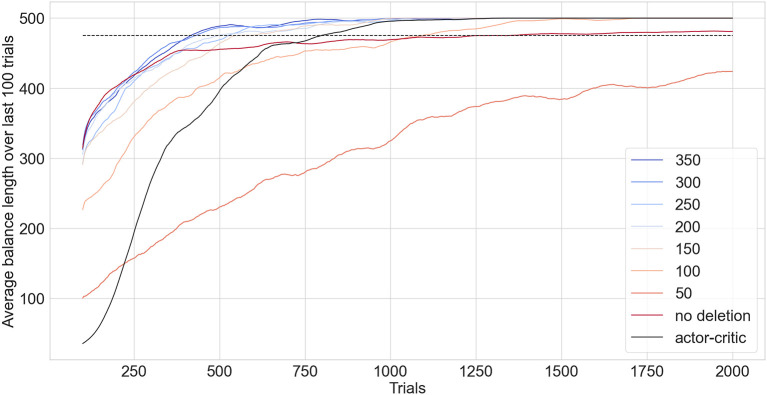
EDN with varied network size limits benchmarked against an ANN trained in tensorflow using an actor-critic model (black) applied to the inverted pendulum task. The lines show the running average of the last 100 trials with the dashed line showing the threshold performance required for the task to be considered solved. Each configuration is repeated 100 times and the average of their performance is shown.

Figure 10 compares the performance of EDN against an ANN trained with GD. The quick acquisition of information enables EDN to reach a stable control of the inverted pendulum almost twice as fast as the GD approach case. The actor-critic model takes on average 798 trials before a stable configuration is reached. The best configuration of EDN, with a maximum network size of 350, was able to solve the task in an average of 415 trials. Similar performance is seen for network sizes of 200 and above. The effect of maximum network size on performance is most noticeable below 150 neurons. At this point neuron deletion becomes too frequent to keep a stable set of behaviors in the network. At 100 neurons the performance significantly suffers with the network taking 1,091 trials to complete the task. A maximum network size of 50 puts strain on the learning with it failing to solve the task in 2,000 trials, only achieving an average balance length of 424 timesteps at the end of training.

Without neuron deletion EDN takes on average 1,250 trials to complete the task. Performance is initially better than the actor-critic, displaying the fast learning capability of EDN, although the convergence to stable balancing proves harder leading to more trials needed to average over 475 over the last 100 trials. During training without neuron deletion it was noticed that there were a number of trials in which the learning did not converge to stable behavior. Instead the performance would quickly drop from balancing for the full duration to struggling to get balance for longer than 100 timesteps. This is likely a result of beneficial behaviors being inhibited *via* neurogenesis during the learning process and, although those actions are useful to balancing, causing strain on further learning. Neuron reinforcement and deletion avoids this pitfall by rewarding neurons which contribute to good behavior and deleting the ones that do not, resulting in a better performing and more condensed network.

The relative speed with which EDN is able to complete this task is a result of the instant labeling of poor behavior and the adapting of performance. This allows a behavioral space to be built in which actions resulting in negative results are avoided. The rewarding and subsequent deletion of neurons enables this further by injecting positive reward into neurons which are contributing to good behavior and removing the neurons which do not aid performance. Overall this incorporates reinforcement learning signals into the network behavior instantly and in a one-shot fashion.

### 3.7. Parametric analysis

Parametric analysis was carried out on the wine classification task. The following section discusses the effect of *s*, *E*_*th*_ and input selection with a focus on the convergence and number of neurons and synapses generated during learning. All experiments used the same random seed and were averaged over a stratified 10 fold cross validation. Unless otherwise specified the parameters used for all test were *s* = 0.4, *E*_*th*_ = 0.2, and *s*_*th*_ = 0.1.

#### 3.7.1. The effect of kernel spread

**Figure 11:**
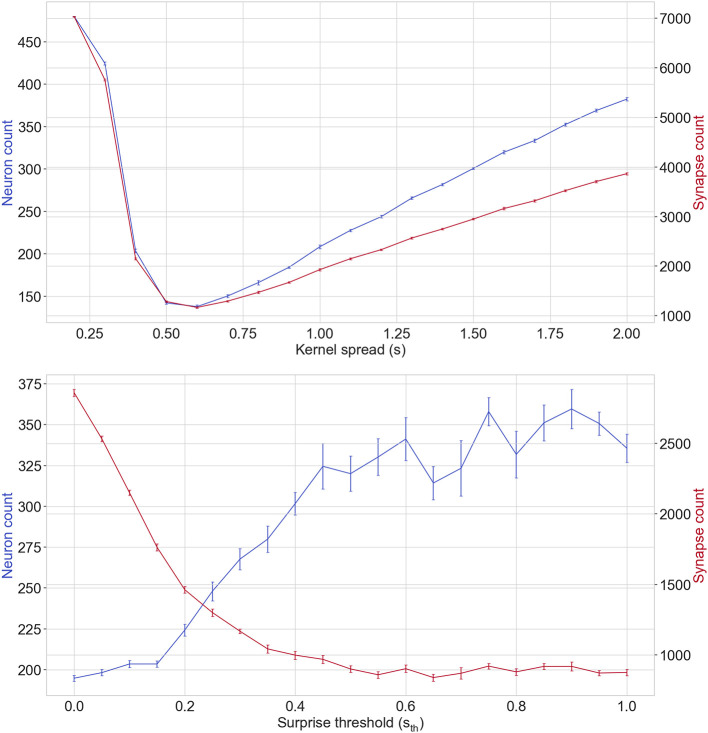
Showing the effect of range of parameter values for kernel spread *s* **(top)** and surprise threshold *s*_*th*_ **(bottom)** on neuron and synapse count after training on the wine classification task. The left y-axis and the line in blue of each plot corresponds with neuron counts. The right y-axis and the red line correspond with the synapse count. The vertical bars show the standard error over a stratified 10 fold cross validation.

The top plot of Figure 11 displays how kernel spread affects the final neuron and synapse count of EDN networks during training. Generally, the more neurons created the worse the performance as the error was more often above *E*_*th*_. Low values of *s* produce many neurons as the hat function is too narrow to allow information transfer across examples. The synaptic response is too specific and therefore the neuron is only active when receiving almost the exact same input causing it to overfit to the training data and perform poorly in testing. When *s* is large there is the opposite problem. Note that all inputs are normalized to fall between 0 and 1 and therefore any *s* > 1 will be active at least by some amount for any input. This means with large values of *s* that there are more neurons active at any one time and, therefore, there are over generalizations made about the data. This hurts performance with testing accuracy being far more erratic, especially during the early stage of learning. However, with further training and neuron generation a balance is found in the network for different output activations bringing the final testing accuracy to a comparable level with more appropriately chosen values of *s*.

In Figure 11, it can be seen that the fewest neurons are created when *s* = 0.6, however, this does not correspond to the best testing accuracy. The best performance was found when *s* was slightly below this level at 0.4; this is likely because there is less transfer of information between saved data points resulting in more triggering of neurogenesis. Increased neurogenesis in tandem with slightly more specific neuron activation leads to better defined input-output mappings. Overall the choice in *s* is a balance between overfitting to the training data at low values and over-generalizing from the training data with high values.

#### 3.7.2. The effect of error threshold

Error threshold, *E*_*th*_, controls the level at which neurogenesis is triggered. When *E*_*th*_ = 0 every training example triggers neurogenesis resulting in as many neurons in the network as examples presented. Increasing the threshold slightly leads to fewer neurons being created with little effect on testing accuracy. It was found that *E*_*th*_ > 0.2 was when performance started to be non-negligibly affected, at this point the model becomes a less complete representation of data and testing accuracy reduced. As *E*_*th*_ increases this effect becomes more pronounced with fewer neurons being saved and accuracy dropping until eventually neurogenesis is no longer triggered and the model remains empty. It is possible that some form of annealing of *E*_*th*_ could allow a detailed model to first be created and then add neurons after that if the magnitude of the error is large helping to reduced network size that remains representative of the data.

#### 3.7.3. The effect of surprise threshold

The method used for selection of synapses in classification tasks is to compare an expected input with the correct input and select the ones with the highest disparity (see Section 2.2.2). The inputs with a surprise value above *s*_*th*_ are chosen to seed the synapses of the newly formed neuron. The bottom plot of Figure 11 shows how varying *s*_*th*_ effects the number of synapses and neurons created after training on the wine classification task.

Similar to the results of *s*, generally speaking the fewer neurons created the better the performance on the task. From the plot it can be seen that lower values of *s*_*th*_ lead to fewer neurons being created and more synapses being created. For *s*_*th*_ < 0.2 the final testing accuracies are equivalent but fewer synapses are created overall the larger the threshold. This shows that the selection process can allow the most important inputs to be selected without hurting overall network performance. This puts focus more on feature-output mappings rather than input-output mappings.

As *s*_*th*_ is increased further the model suffers as the inputs selected by the model becomes too fragmented and no longer represent the data as a whole. This leads to fewer synapses being created overall whilst the neuron count increases. For high values of *s*_*th*_ eventually a point is reached at which neurogenesis is triggered and no input synapses are selected for the new neuron causing the model to stagnate. Generally the choice of *s*_*th*_ is a balance between saving all information and extracting a reduced form. From MNIST experiments it was found *s*_*th*_ = 0.4 allowed the most important features to be extracted resulting in the best performance, again, putting emphasis on the feature-output mapping rather than the input-output mapping.

#### 3.7.4. How random input selection size effects performance

An alternative method for input selection is to select *n* inputs randomly, without replacement, to form the synapses of the new neuron. This avoids the need for constructing an expected input and comparing it with the actual input, reducing the computational overhead for each training example. It was found that when the number of synapses randomly selected was increased the testing accuracy generally increased, however, this also led to an increased neurogenesis in these experiments. It would be expected that the better the testing accuracy the less neurogenesis, however, neurogenesis is triggered by the magnitude of the error produced not the classification. The speed at which the error decreased was uniform across the values of *n* but the larger the value of *n* the larger the error which means more instances in which neurogenesis was triggered. A likely cause for this is that an increased number of synapses per neuron leads to more accurate but less precise representations for each class (accuracy being a measure of correctness and precision a measure of exactness). A more accurate representation of input-output mappings can be stored on the neuron with more synapses as a more complete representation is stored of a data point. However, reducing the number of synapses stored on a neuron makes the input-output mapping more precise as the number of number of inputs involved in an output prediction are limited. This precise representation comes at the cost of a more fragmented model of each class which hurts testing accuracy. This explains why the MNIST experiments run with random input selection did not get the best performance when all synapses are selected. The best performance does not come from complete input-output mappings but extracting the most important feature-output mappings, creating a more precise representation of the classes.

## 4. Discussion

This work has outlined how non-linear synaptic activations can be used in conjunction with neurogenesis to tackle a range of tasks in an online fashion. A new neuron is created following an error and the synapses are used to store input values related to that error. This can be done in parallel with network operation and requires no further updating of parameters which means previously stored information is never forgotten from the model. The instant incorporation of information into the network enables behavior to be updated immediately. In a robotic scenario this could enable an agent to explore and acquire information without the need for multiple trials and offline computation. This would allow robots to explore unknown environments whilst updating models of their surroundings. It could also be applied to adjusting their behavior to account for damage to components without the need for outside intervention, much in the same way humans limp following an injury.

EDN has been applied to a range of tasks (classification, regression and reinforcement learning) and a fast and data efficient learning has been displayed. EDN offered a speed up of 25 times on the wine classification task, 135 times on the auto-mpg regression task, 2 times on MNIST digit recognition, and 2 times on the inverted pendulum reinforcement learning task compared to a traditional ANN trained with GD. This improvement in speed is a result of the encoding of input and output values in the neurons enabling immediate application of new information following error driven neurogenesis without the need for gradual parameter updates. Classification accuracy was the same for both EDN and GD, however, mean squared error for the regression task was three times smaller with EDN compared to GD. EDN was able to readily attach positions in the input space to regression values, this led to both faster and more accurate performance compared to gradient descent. This is likely a consequence of the non-linear properties of the regression task making capturing the exact input-output mapping more difficult for traditional ANNs. Gradient descent with ANNs requires the building of a statistical summary of the data and encoding it in the weights of a predefined network, paired with the neurons producing an output relative to the distance from a hyperplane (see Section 2.1.2) this makes producing a smooth non-linear output value difficult.

MNIST presented the greatest challenge for EDN with the main gains being shown in comparison to GD in terms of speed. The power of EDN comes from its ability to store a value on the synapses and attribute a particular output to it through the neuron, following this any input which is similar will cause the neuron to activate the associated output. MNIST provides a challenge to this as members of the same class could have exactly the same inputs with only a slight spatial transformation. The result of this transformation is that the input values will now be different, and although the general structure of the input is the same as a previously saved input the input-output mappings will no longer be helpful. If the synapses or neurons could encapsulate possible input transforms, effectively giving the synaptic triangle kernel a spatial spread, then there could be more information transfer between examples. Possibly some form of convolutional filter may achieve a similar affect.

Another possible reason for the difficulty in reaching higher levels of performance on MNIST is because of the increased dimensionality of the input. This makes sampling the correct features and using them to classify inputs increasingly difficult. It was shown that altering the way in which the inputs are sampled can effect the performance of EDN, if sampling or the generation of expected response can be improved it would increase the performance of the model further. A potential route for this could be the implementation of an attention mechanism that adaptively selects different inputs rather than relying on a collective representation of individual classes. It is also possible that a neuron deletion dynamic, as was displayed in reinforcement learning, could enable EDN to remove neurons whose activity does not aid performance in classification.

In the reinforcement learning task the positive reward generated at each timestep was fed into the network and enabled the deletion of the least useful neurons. This acted to put a cap on the network size but came with the added benefit of improving the speed of task completion by removing the least beneficial input-output mappings. In future work it could be useful to find an analogous mechanism for other learning regimes by which network growth can be restricted and potentially, in parallel, improve the performance of the model. As neurogenesis is triggered by errors if they cannot be brought below threshold then network size continues to increase. Some form of early stopping or error threshold annealing may also help curtail the continual growth of the network as performance generally converges quickly with only minor fine tuning happening afterwards even though network growth continues.

The mechanism by which information is stored within the network allows easy access to what specifically is influencing behavior leading to more informed model debugging. Saved data can also be extracted for post processing and the creation of a more condensed model. This is of particular importance given the size to which EDN can grow a network. Without a cap on network size the number of neurons continues to grow in the presence of error, therefore, a mechanism by which saved data can be elucidated into a reduced model would allow learning over a much longer time period without a growing memory footprint. This would also enable offline training without the need for further examples as previous inputs are saved within the model and can be extracted. The condensed model may also provide beneficial extra dimensions to the inputs, akin to the kernel trick used in support vector machines (SVM). In SVMs, kernels are used to provide additional dimensions to the data thereby allowing a linear classifier to separate the data. If the produced EDN model could be used to generate ANN neurons this would add extra dimensions to the data which future learning with EDN can take advantage of.

If the branching structure of dendrites could also be made a part of the network's connections, more complex dependencies between features could be constructed. As was shown in the results the best performance came when the most appropriate feature-output mappings were captured by the network and not solely the input-output mappings. Branching dendrites could allow a richer description of inputs to be represented by a single neuron as activity would no longer be a uniform sum of synapse activations. This may allow each neuron to have branching dependencies of inputs with some being more important than others. If neurons could also be allowed to connect to other hidden neurons, instead of only inputs, it would enable synapses to be responsive to higher level features and allow more complex topologies to grow.

EDN updates its model by adding new neurons to its current network in response to errors. GD updates its model by moving the weights in the direction gradient calculations dictate will produce less error, eventually creating a statistical summary of the data in its weights. This puts the model bias in EDN on the order in which information is presented as opposed to bias coming from the random initialization of the network as in GD. This may be utilized with a form of curriculum learning in which simple and representative examples are first presented and the difficulty is increased from there. This is similar to how we teach children, first starting with simple concepts and characteristic features then incrementally building from there. Modern GD approaches do not require this as they can eventually build a statistical summary of the input-output mappings without concern for first capturing the fundamentals.

This work explores how neurogenesis in tandem with synapse/dendrite non-linearities can be used to store information about inputs in a form that can be used to alleviate errors. It enables a fast acquisition of information and updating of a behavioral model. Ultimately, this is not a panacea and is only a part of the puzzle. Traditional ANN transfer functions, whose activity is relative to the distance from a hyperplane, allow neurons to be active beyond a local area of the input space, providing broader statements about input-output mappings. It is likely that a hybrid approach between quick, functional data acquisition, and the building of general statistical representations of data is required to create more complete learning systems. This could allow the data gathered within the day to be condensed down into a reduced model, iteratively increasing the complexity of the representations captured by the network. Something akin to this may happen in biological brains where wakeful hours are used to acquire data through interacting with the world, followed by sleep in which information is consolidated and pushed into a more general model whilst freeing up previously used neural real estate.

## Data availability statement

The original contributions presented in the study are included in the article/supplementary material, further inquiries can be directed to the corresponding author/s.

## Author contributions

The research within was conducted by AP with guidance and proof reading from OR and SF. All authors contributed to the article and approved the submitted version.

## Funding

This work was supported by the EPSRC Manchester Centre for Doctoral Training in Computer Science (EP/IO28099/1) and EU ICT Flagship Human Brain Project (H2020 785907 and 945539).

## Conflict of interest

The authors declare that the research was conducted in the absence of any commercial or financial relationships that could be construed as a potential conflict of interest.

## Publisher's note

All claims expressed in this article are solely those of the authors and do not necessarily represent those of their affiliated organizations, or those of the publisher, the editors and the reviewers. Any product that may be evaluated in this article, or claim that may be made by its manufacturer, is not guaranteed or endorsed by the publisher.
